# Beyond the Tip of the Iceberg: Using Systems Archetypes to Understand Common and Recurring Issues in Sports Coaching

**DOI:** 10.3389/fspor.2019.00049

**Published:** 2019-10-16

**Authors:** Scott McLean, Gemma J. M. Read, Adam Hulme, Karl Dodd, Adam D. Gorman, Colin Solomon, Paul M. Salmon

**Affiliations:** ^1^Centre for Human Factors and Sociotechnical Systems, University of the Sunshine Coast, Sippy Downs, QLD, Australia; ^2^School of Exercise and Nutrition Sciences, Queensland University of Technology, Brisbane, QLD, Australia; ^3^School of Health and Sports Sciences, University of the Sunshine Coast, Sippy Downs, QLD, Australia

**Keywords:** system thinking, coaching, performance, system archetypes, talent development

## Abstract

**Background:** Systems thinking, a fundamental approach for understanding complexity, is beginning to gain traction in sports science. Systems archetypes (SAs) describe common recurring patterns of system behaviors and have been used extensively in other domains to explain the system wide influences on behavior. SAs look at the deeper levels of systemic structure by identifying what creates system behaviors, which supports the development of interventions to identify and resolve problem sources.

**Methods:** Four commonly used SAs were used to explain the dynamics underpinning recurring issues for coaching in football: (1) Fixes that fail, (2) Shifting the burden, (3) Drifting goals, and (4) Success to the successful. The SAs models were built, refined and validated by seven subject matter experts (SMEs) including experienced football researchers, systems thinking experts, an international football coach, a skill acquisition specialist, and an experienced exercise scientist.

**Results:** The findings show that the SAs fit well in the football coaching context, providing further evidence that a complex system thinking approach is required when considering football performance and its optimization. The developed SAs identify the factors that play a role in recurring issues in football coaching and highlight the systemic structures that contribute to the issues. The developed SAs identify the appropriate leverage points in the system where sustainable change can be made to improve coaching practice and subsequent performance of players.

**Discussion:** A common theme emerging across the analyses was that systemic problems often arise in football when quick fixes are attempted. Whereas, improvements to system behavior usually require a delay after the implementation of the appropriate corrective action. The SAs developed in the current study also provide practical templates of common problems in football that can be used to prompt discussions around how to avoid ineffective interventions and instead make sustainable improvements across multiple aspects of football performance.

## Introduction

In many domains outside of sport, including business, economics, engineering, and environmental sustainability, systems thinking has been adopted as an alternative approach for understanding and responding to complex problems (Senge, 1990; Bosch et al., 2007; Cabrera et al., 2008; Nguyen and Bosch, 2013). Systems thinking provides an holistic view of behavior and system performance, and an understanding of how system components interact and influence one another through non-linear feedback, and causality between system components (Senge, 1990; Sterman, 2006; Dekker, 2011). The primary tenet of systems thinking is that behavior in any context cannot be understood by examining components in isolation; rather, the system as a whole should represent the unit of analysis (Ottino, 2003). The approach has its roots in general systems theory and complexity theory (von Bertalanffy, 1969; Skyttner, 2005), and provides various models and methods designed to support systems analysis and design. It has been argued that human beings are innate systems thinkers (Senge, 1990). This is supported by research demonstrating the capabilities of young children to rapidly grasp and excel at systems thinking tasks (Clark et al., 2017). However, it is our learning environments that potentially stifle this innate ability by teaching us that to understand complex systems we should break them apart into manageable isolated components, understand how the components work, then reassemble them in an attempt to understand the system as a whole (Senge, 1990; Meadows, 2008). This approach to solving complex issues has its origins in the scientific revolution where a mechanistic Newtonian-Cartesian world view was developed, and continues today (Dekker, 2011). However, this reductionist approach has been widely criticized by systems thinkers as it often fails to consider the multiple components within a system, how the components interact dynamically, and the resulting emergent properties of these interactions (Cilliers, 1999). As a result, it is difficult to fully understand a given systems' dynamics, or its potential behaviors. In sport, an analogous example would be to conduct an in-depth analysis of individual football players without considering how those players interact as part of a system of other players, their coaches, and support staff. This reductionist approach misses the bigger picture and overlooks the key concept of systems theory which is the interdependency of system components (Senge, 1990). Without these influencing relationships the system becomes a collection of unrelated autonomous components (i.e., not a system at all).

There is an increasing amount of scientific literature which makes the argument that sport systems are complex in nature (Hulme et al., 2018b). Sports systems have been shown to possess many of the accepted characteristics of complex systems (Cilliers, 1999), including multiple components, non-linear interactions, emergent properties, dynamism, re-currant feedback loops, path dependence, and ignorance of components. Building on this, complex systems analyses have recently been applied in sport to injury prevention (Bittencourt et al., 2016; Hulme et al., 2018a), performance and performance analysis (Duarte et al., 2012; McLean et al., 2017, 2019), and sports science generally (Soltanzadeh and Mooney, 2016; Mooney et al., 2017). As a result, systems approaches in sport research have created new knowledge, however, further work is required to translate these into practice. Despite the growing number of systems-based analyses in sport, sporting performance is typically analyzed by studying components of performance in isolation (Bishop, 2008; McLean et al., 2017). This has involved isolating components of the whole system into manageable sub-disciplines such as physiology, biomechanics, and psychology. Although these disciplines are useful for understanding specific aspects of athlete behavior, the variables studied within these disciplines do not operate in isolation, which highlights the necessity to investigate the interactions between variables. Moreover, an understanding of behavior in complex systems can only be achieved by taking the overall system as the unit of analysis (Ottino, 2003). Notational analysis is a pertinent example of how sports scientists have fixated on decomposing sports team and player performances into isolated components such as examining passing, tackling, and running as isolated events (O'Donoghue et al., 2008; Rampinini et al., 2009). This common approach in sport science overlooks the interdependent relationships of the system components which are the defining elements of system behavior (Hulme and Finch, 2015; McLean et al., 2017, 2019; Hulme et al., 2018a). The result of this independent sub-discipline analysis is a detailed understanding of each isolated component, but a limited understanding of the interactions of systems components. A systems approach acknowledges that the whole system is much greater than the sum of its individual components of sports performance (Hulme et al., 2017). For example, sports team performance cannot be understood by isolated analyses of individual players, it is the interactions between the players that create performance (Duarte et al., 2012; Travassos et al., 2013). As such, a comprehensive understanding is required to fully appreciate the interactions and emergent properties that underpin performance, and the extent to which those interactions influence behavior. Understanding systems thinking in football is to acknowledge that there are multiple levels of analysis. For example, the athlete, the match, the team, the club, the league and the governing bodies can all be viewed and analyzed as independent systems. The use of systems thinking-based methods and tools is one approach that could be used to address this knowledge gap.

The aim of this article is to introduce and demonstrate systems thinking in the context of the complexity of coaching in football (Partington and Cushion, 2013), across multiple levels of system analyses. In addition, the current article will apply system thinking tools to identify the appropriate leverage points which can enact change in system behavior, and subsequently to demonstrate the capability of systems thinking to understand and potentially resolve issues seen in football coaching.

### The System Thinking “Iceberg” Model

**Figure 1:**
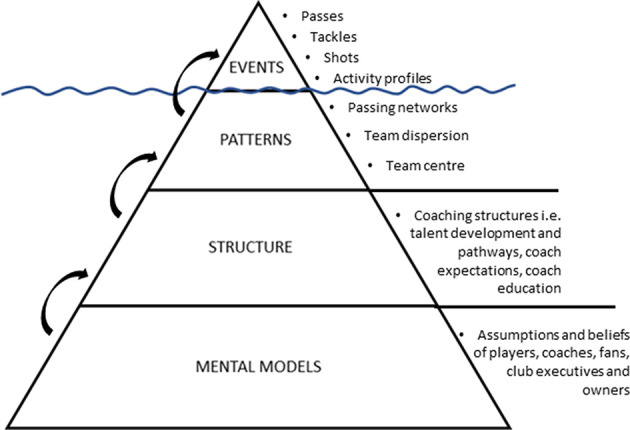
Conceptual systems thinking “iceberg” representation of system levels. Adapted from Senge (1990). Arrows illustrate how the lower levels of the iceberg generate the upper levels of the iceberg. Example components of the four system levels are presented.

A simple conceptual view commonly used for understanding systems thinking depicts the system as an iceberg (Figure 1) (Senge, 1990; Maani and Cavana, 2007). The system thinking iceberg stems from organizational management (Kim, 1994; Braun, 2002) and can be used to explain how management and policy actions commonly manifest (Kim, 1994). The levels of the iceberg are interrelated, for example, the mental models of key stakeholders within the system determine how the system is structured, which then generates system patterns which generates system events (Senge, 1990; Kim, 1994). For example, the tip of the iceberg visible above the water represents the *events* in a given system, the second level lying just beneath the surface represents the systemic *patterns*, and the deepest and largest levels represents the systemic *structure* and *mental models* of people within the system (Figure 1) (Senge, 1990; Maani and Cavana, 2007). The system events are what we see and notice, the patterns are a series of less noticed phenomena that when performed together interact to create events. The systemic structure of the iceberg represents how the system is organized (in terms of physical, social, and regulatory structures). The model suggests that it is the system structure which generates the patterns and events. The mental model level of the iceberg represents the assumptions, beliefs, and values that shape and perpetuate the system structures (Senge, 1990). It is argued that the mental models of the system stakeholders are often different and can conflict (Leveson, 2004).

In sport, it can be argued that the focus on discrete and isolated events has enabled an in-depth understanding of the tip of the iceberg, and of isolated system events. It is therefore at the deeper levels of the iceberg where our understanding of sport performance can potentially be advanced. A detailed understanding of the system structure and mental models will provide information on the behavior of the system, why recurring problems exist, and how issues can be resolved to improve performance.

### Systems Thinking in Coaching

Match performance analysis which guides coaching decisions in sport (Wright et al., 2014; Sarmento et al., 2017) is a discipline primarily focused on events, such as what occurred, where it occurred, who performed it, and when it occurred. Within the past decade there has been an increase in research on the patterns in football, for example group behaviors such as team dispersion, and passing network analyses (Sarmento et al., 2017). However, it is apparent that there is a lack of understanding of how systemic structures and mental models interact to generate such events and patterns. Focusing on isolated events can lead to coaches making event-driven decisions that do not necessarily provide an holistic view of the specific decisions made. For example, a coach may perceive that a player is not performing as expected, based on their activity profile or how many unsuccessful passes they made, which can provide a misleading view of performance driven by analysis of discrete match actions (i.e., events). Without the context of understanding the system wide influences on player behaviors, it may not be possible to fully understand how and why these actions were created by the player at the time. For example, in other disciplines such as safety science, there has been a paradigm shift away from attributing individual blame toward attempts to understand the myriad of systems wide factors that influence behavior (Rasmussen, 1997; Dekker, 2011; Salmon and Read, 2019). Sport science can learn from this example, by better understanding the multiple levels of the sports system (Hulme et al., 2018a), allowing more information to be obtained regarding why and how the events occurred. Furthermore, a systems thinking approach argues that making changes to the structure of the system will have a far greater influence on improving events than decisions that are made at the superficial events level (Meadows, 1999).

### Causal Loops

**Figure 2:**
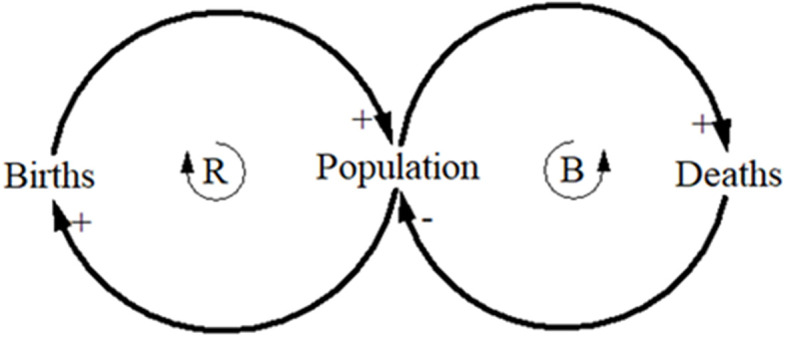
Casual loop diagrams representing the reinforcing loop (R) of births on the population, and the balancing loop (B) of deaths on the population. + indicates a positive influence on the variable, and – indicates a negative influence on the variable.

Causal loops are the building blocks of system thinking (Senge, 1990). All systems comprise interacting networks of reinforcing (positive) and balancing (negative) feedback loops that influence system behavior (Sterman, 2000). Reinforcing loops (or positive feedback) are actions that afford change in one direction to produce even more change in the same direction, whereas, balancing loops (or negative feedback) work to keep the system in a state of equilibrium. Balancing loops resist change in one direction by producing change in the opposite direction (Senge, 1990; Sterman, 2000). Causal loop diagrams (CLDs) provide a method to represent these dynamic interrelations via visual representation which assists in communicating the complexity of a given system (Senge, 1990; Kim, 1993). CLDs comprise variables connected by arrows which depict the causal influences between the variables (Sterman, 2000). This is opposed to a unidirectional cause and effect view where the interaction between variables is assumed to be linear (e.g., A causes B causes C causes D), which assumes that events occur sequentially (Kim, 1994). This linear thinking approach does not consider feedback between variables. For example, variable A may cause B which may cause C which may then feedback to influence the behavior of A or B in a different way. Given the limitations of linear thinking, CLDs are useful for capturing hypotheses about causal dynamics, for eliciting and capturing mental models, and for communicating the feedback mechanisms that may be responsible for a particular problem (Sterman, 2000). The simple example of the influence of births and deaths on population in Figure 2, demonstrates how births increase the population which further increases births (R1), while an increasing population will increase death rates which in turn will work to resist the increasing population (B1).

### Behavior Over Time Graphs

**Figure 3:**
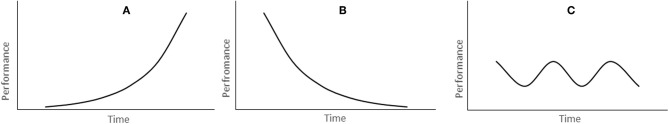
Conceptual behavior over time graphs. **(A,B)** Represent reinforcing feedback loops, and **(C)** represents a balancing feedback loop.

Behavior over time graphs are used in system thinking approaches to focus on patterns of change over time rather than focusing on specific events. Understanding how a system behaves over time provides an understanding of the interrelated and dynamic relationships among the variables in a system (Kim, 1993). Behavior over time graphs can also be used to identify which types of system processes are occurring. For example, a rapidly increasing or decreasing behavior over time graph indicates that reinforcing loops are influencing the system (Figures 3A,B), because they reinforce change in a certain direction. In contrast, an oscillating behavior over time graph would indicate that balancing feedback mechanisms are occurring in the system (Figure 3C), because they are trying to change a certain system behavior. Conceptual behavior over time graphs are shown throughout this article to explain the relationships between variables of the specific coaching issues discussed.

### Leverage

Leverage points are places within systems where a small change can be made that has large scale changes on the entire system (Meadows, 1999). An advantage of systems thinking over linear thinking is that it can often be used to reveal the most appropriate place within the system structure to enact change via leverage points. All system structures contain leverage points, and systemic change can be accomplished by finding the appropriate leverage point to apply interventions (Meadows, 1999). All systems resist attempts to change their behavior, and a common issue for linear thinkers when identifying leverage points is that they are often counterintuitive to the goals of the system (Senge, 1990; Meadows, 1999). Leverage points can be as simple as adding constraints or parameters into the system, however, changing mindsets (mental models) and paradigms are the most effective places to intervene with leverage points (Meadows, 1999). Despite this, systems change is difficult to predict and often differs from expected results and desired outcomes (Sterman, 2000; Friedman, 2004), which further highlights the counterintuitive nature of appropriate leverage points. A valuable method for identifying the most appropriate location for leverage in order to change the behavior of the system is systems archetypes.

### Systems Archetypes

Using CLDs and behavior over time graphs as their basis, SAs are generic system templates that describe and classify the structures of system behavior over time (Paich, 1985; Graham, 1988; Senge, 1990; Kim, 1993; Maani and Cavana, 2007). The development of generic templates of system behavior is not new. For example, pioneering system scientists from the Massachusetts Institute of Technology (MIT) have identified and applied generic system models to complex systems issues since the 1950's (Forrester, 1958; Graham, 1988). SAs describe common recurring patterns of systemic behaviors and have been used extensively in many domains such as business, economics, and ecology to explain system behavior and systemic issues (Sterman, 2000; Nguyen and Bosch, 2013). Although experienced practitioners may already be aware of recurring issues, they are not always aware of how to explain the dynamic system of interacting factors. Further, they are not always aware of the factors that interact to create them, or how to identify appropriate leverage points to enact change (Senge, 1990).

SAs can be used to respond to these knowledge gaps by investigating the deeper levels of the system structure to identify what generates system behaviors, which allows for interventions to target problem sources. Because of the capability of SAs to make system structures explicit, they can be used as (1) *diagnostic tools*; to understand systemic issues and to identify why given issues are occurring, (2) *proactive planning tools*; rather than simply diagnosing issues it is possible to identify the structure of systemic issues and act to reduce or remove them in the future, and (3) *theory building tools*; SAs can help to build theories fundamental to rethinking future systems (Kim, 1994). While there are multiple SAs within the literature (Graham, 1988), there are a set of eight popular and commonly used SAs that represent multiple scenarios: (1) Fixes that fail, (2) Drifting goals, (3) Growth and underinvestment, (4) Shifting the burden, (5) Success to the successful, (6) Tragedy of the commons, (7) Limits to success, and (8) Escalation (Senge, 1990; Kim, 1993; Maani and Cavana, 2007). SAs can be applied at multiple levels within sport including teams, clubs, and entire organizations or federations to understand performance.

## Methods

### Constructing the System Archetypes

To identify SAs, CLDs are used first to depict the overall system variables within a pre-determined boundary, which in this case was coaching in football. The purpose of CLDs is to identify system variables and visualize the reinforcing and balancing influences between them (Nguyen and Bosch, 2013). CLDs are typically developed using a group model building process (Sterman, 2000; Bérard, 2010). In the current study, two experienced football and systems researchers (Salmon et al., 2009; McLean et al., 2017), and an international senior level men's football coach were involved in the modeling process. The three SMEs have published multiple peer reviewed research articles on football, and two coach football professionally. The use of subject matter experts (SMEs) to engage in group modeling processes is common in a wide range of systems analyses research (Naikar, 2013; Read et al., 2016), including in sport (Morris and O'Connor, 2016; McLean et al., 2017, 2019). The first step involved identifying common recurring issues in football coaching. This involved drawing upon multiple data sources, including peer reviewed literature, SME experience in football research and practice, media articles, and publicly available information to identify common problems in coaching in football. Four broad issues were identified (1) a high turnover of coaches, (2) skill development in youth players, (3) national coaching curriculums, and (4) youth player selection procedures. Each of the identified issues were represented as CLDs where dynamics of the problem were conceptualized to communicate the important feedback loops. The CLDs were used to make hypotheses about the system variables by examining the reinforcing and balancing influences. From the four developed CLDs, the SMEs identified characteristic system behaviors that aligned with the structure of existing generic SAs: (1) Fixes that fail, (2) Shifting the burden, (3) Drifting goals, and (4) Success to the successful. Each SA produced by the SMEs was subsequently reviewed by two additional systems thinking experts (Salmon et al., 2016; Hulme et al., 2018a), a skill acquisition expert (Gorman and Maloney, 2016), and an experienced exercise scientist (Kerherve and Solomon, 2018). The review process was used to determine the appropriateness of the identified problem to the specific archetype, and the terms used within the archetypes. The archetypes were refined by three members of the research team based upon appropriate suggested revisions from the SME review process. The four developed SAs are discussed.

### Systems Archetypes Applied to Coaching in Football

#### Fixes That Fail

**Figure 4:**
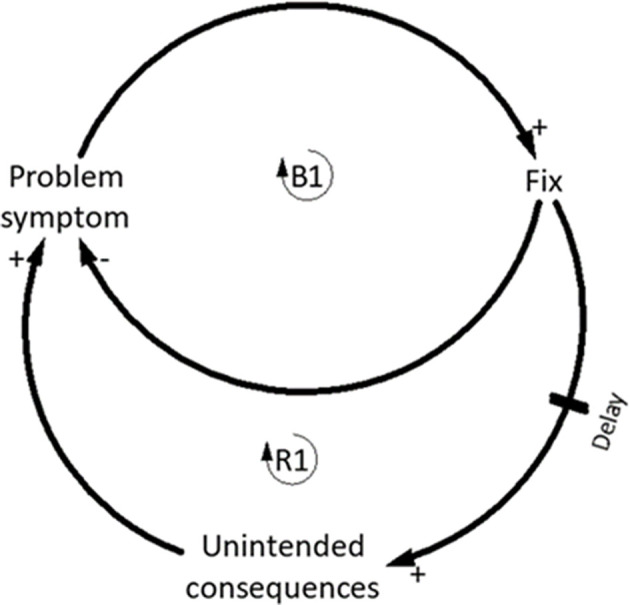
Generic fixes that fail template. **/** indicates a delay in the system. Adapted from Braun (2002).

Fixes that fail is characterized by one balancing loop and one reinforcing loop (Figure 4) and occurs when a problem symptom requires a fundamental change in order for it to be a fixed. Instead, a quick fix is applied and whilst the problem symptom is temporarily alleviated (B1), unintended consequences emerge and the problem symptom either returns (R1), or worsens (Senge, 1990; Kim, 1993) (Figure 4). Fixes that fail is commonly used diagnostically to identify recurring issues, by developing an understanding of the system structure contributing to specific situations (Kim, 1994).

**Figure 5:**
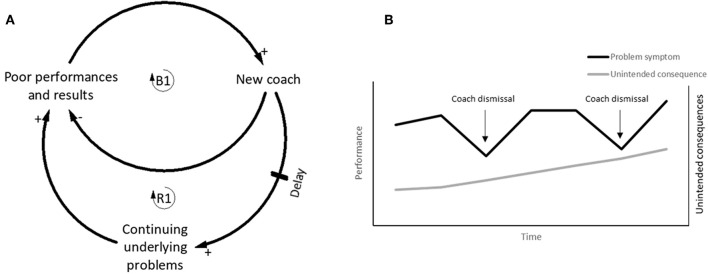
**(A)** The Balancing loop (B1) shows the fix to the problem symptom, by appointing a new coach which temporarily improves performance. The reinforcing loop (R1) shows the continuing underlying problems at the club are degrading performance and results. **(B)** The conceptual behavior over time graph showing the drops in performance are alleviated by the appointment of a new coach yet plateau before eventually dropping again and the cycle continues due to the constantly increasing unintended consequences. Adapted from Braun (2002).

The Fixes that fail archetype is often seen in football with the in-season removal of coaches following poor team performance and results. A study of 42 coach dismissals across 14 successive seasons of the top Dutch football league, found that coaches are often removed and replaced following a run of poor performances and/or results (van Ours and van Tuijl, 2016). The immediate fix is often to remove the current coach and appoint a new coach, which in the short term has been shown to alleviate the problem symptom by improving results initially (de Dios Tena and Forrest, 2007). Similarly, team performance post-dismissal has been found to be superior to pre-dismissal performance (van Ours and van Tuijl, 2016). However, despite these initial short term improvements, changing coaches in-season has been shown to either have an overall negative net effect on team performance across a season (de Dios Tena and Forrest, 2007; Flores et al., 2012), or no positive performance effects for the clubs dismissing coaches (van Ours and van Tuijl, 2016). The Fixes that fail archetype depicts a representation of this common scenario in football (Figure 5A). Research has also shown that teams having a poor run of form whilst affording their coach time to improve the situation will also have improved results, but the overall negative net effect of not dismissing the coach is reduced (Bruinshoofd and Ter Weel, 2003; van Ours and van Tuijl, 2016).

This highlights that an events-driven decision-making process based primarily upon short term results and performances is a potentially inappropriate course of action. Several reasons have been reported for coach dismissals, most of which eventuate when looking for a quick fix to alleviate the problem symptom (Flores et al., 2012; van Ours and van Tuijl, 2016), which is driven by pressure from sponsors, fans, and media, conflict with players, staff and board members, and the notion that doing something is better than doing nothing (Flores et al., 2012; van Ours and van Tuijl, 2016).

#### Breaking the Fixes That Fail Cycle

The solution to break the Fixes that fail cycle in regard to coach replacements, is to acknowledge that the sacking and hiring of coaches on the basis of short-term poor team performance alone is likely to only temporarily alleviate symptoms. A commitment to solve the underlying cause of the problem is required. For example, time and resources may need to be allocated to identify and implement solutions addressing the fundamental problem (Senge, 1990; Kim, 1993; Braun, 2002).

For clubs to commit to fixing the fundamental problems rather than applying quick fixes, leverage needs to be applied in order to shift the mental models of the club executives, board members, sponsors, fans, etc., to understand that solving the fundamental problem is a long-term strategy. Clubs which regularly dismiss coaches after a run of bad results are often caught in a constant cycle of solving initial problems only to create problems in the future (Senge, 1990; Kim, 1993). In Figure 5B, the behavior over time shows the problem symptom is temporarily alleviated by appointing new coaches, yet the underlying unintended consequences remain unresolved and can even increase.

#### Shifting the Burden

**Figure 6:**
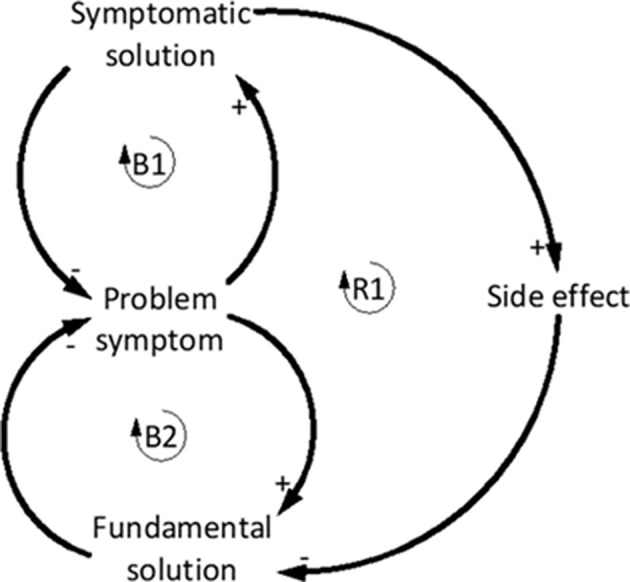
Generic shifting the burden archetype. Adapted from Braun (2002).

The Shifting the burden archetype is also focused on how systems deal with problem symptoms. The structure of this archetype is comprised of two balancing loops and a reinforcing loop where both balancing loops are working to correct the problem symptom. This SA is seen when a problem is addressed by applying a symptomatic solution (B1), which acts to divert attention from fundamental solutions (B2) (Kim, 1993; Braun, 2002) (Figure 6). Shifting the burden is often used as a diagnostic tool, as it supports attempts to understand a problem in order to generate appropriate interventions (Kim, 1994).

**Figure 7:**
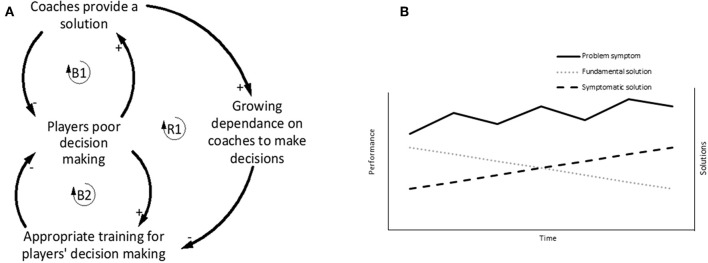
**(A)** Balancing loop (B1) indicates that by providing solutions to problems, the coach reduces the incidence of players making poor decisions. A side effect of this coaching style is that players become dependent on the coach making decisions which can hamper the capability of the players to improve their decision-making (R1), whereas if the appropriate training was delivered, players' decision making is more likely to benefit (B2). **(B)** Conceptual behavior over time graph shows how continually applying a symptomatic solution only temporarily improves performance and the fundamental solutions continues to degrade over time. Adapted from Braun (2002).

In youth football coaching, the Shifting the burden archetype can be used unintentionally by coaches, which subsequently stifles the development of player expertise. The problem symptom demonstrated (Figure 7A) relates to players making poor decisions. To quickly fix the problem symptom coaches begin to make decisions for the players, either by direct verbal instruction, or by using isolated practice drills with continuous and direct instruction (Cushion, 2010; Light et al., 2014; O'Connor et al., 2017, 2018). On the surface, making decisions for the players enables training to flow better, looks good to the onlooking parents, and provides the coach with a sense of control over the training environment (Potrac et al., 2007). However, this approach can encourage the players to become reliant upon the coach to make decisions, which may have negative consequences in game situations where the players must make decisions independently in the absence of the coach (R1). Instead, a degree of variability via game-based training should be encouraged because this approach allows players to learn from their mistakes (Partington and Cushion, 2013). The side-effect of coaches applying symptomatic solutions is that players become dependent on not having to make decisions, which potentially increases poor decision making in matches (Ford et al., 2010).

#### Breaking the Shifting the Burden Cycle

Where this cycle is present in coaching, a shift in mental models is required to understand that an appropriate degree of variability in training and the opportunity to learn from mistakes can enhance the decision-making ability of players (Partington and Cushion, 2013). In the context of the iceberg model, a shift in mental models of the coaches would change the way a coach structures training practice, which will generate different behavioral patterns and events by the players. Coaches may shift the burden because it produces short term results, is easier than having a “messy” training session, or they may not have the skills required to address the problem symptom (Partington and Cushion, 2013). Often the reasons behind the behavior demonstrated by this archetype are well-intended. However, continually applying a symptomatic solution will cause the fundamental solution to degrade over time as seen in the behavior over time graph (Figure 7B) and reduce the capability of the players to develop their expertise (Ford et al., 2010).

#### Drifting Goals

**Figure 8:**
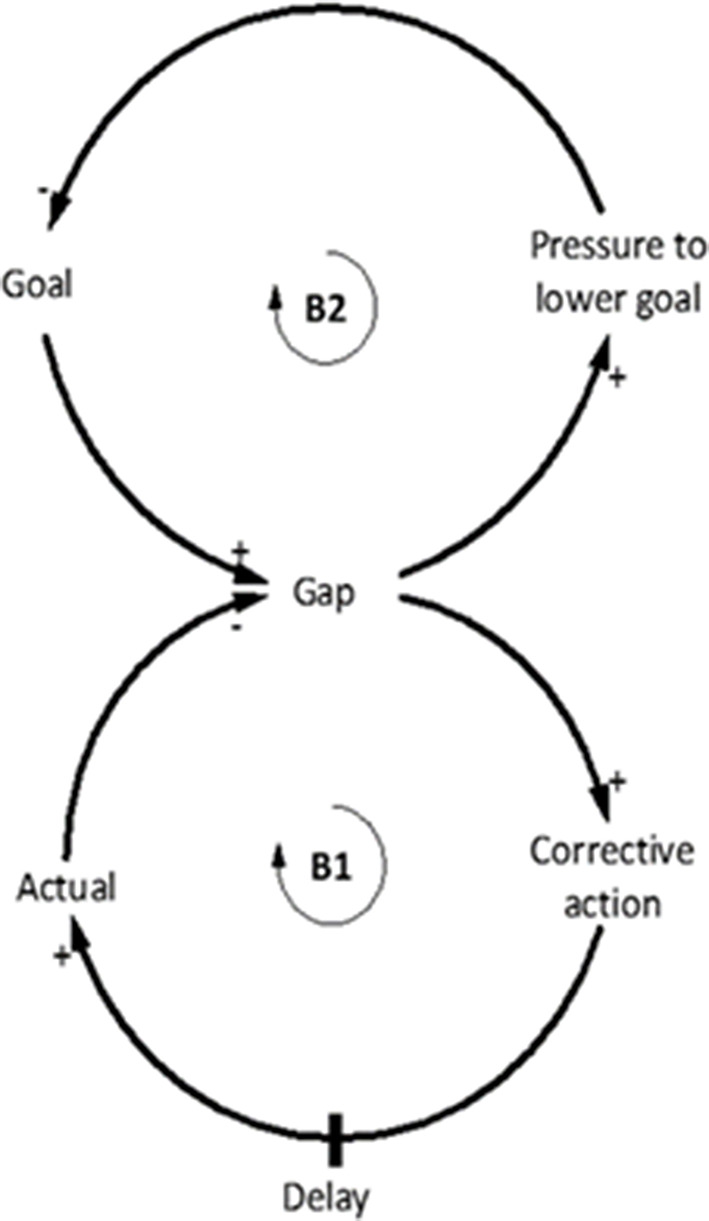
Generic Drifting goals archetype. **/** indicates a delay in the system. Adapted from Braun (2002).

The Drifting goals archetype occurs when there is a gap between the intended goal of the system and actual performance of the system (which requires corrective action to be taken to return the system to the intended goal) (B1) (Figure 8). Typically, there is pressure to apply a quick fix to align the system's goal with actual performance of the system. A common quick fix to reduce the gap between actual system performance and the intended goal is to lower the intended goal, which allows the goal to be more easily achieved (B2), when in fact, lowering the intended goal degrades overall performance (Braun, 2002). The appropriate corrective action is often not a quick fix and requires a delay to bring the current state of the system toward the intended goal (Figure 8). The Drifting goals archetype is classified as a theory building archetype as it attempts to rethink how the structure of the system could be enhanced so that the intended goal does not have to be lowered (Kim, 1994).

**Figure 9:**
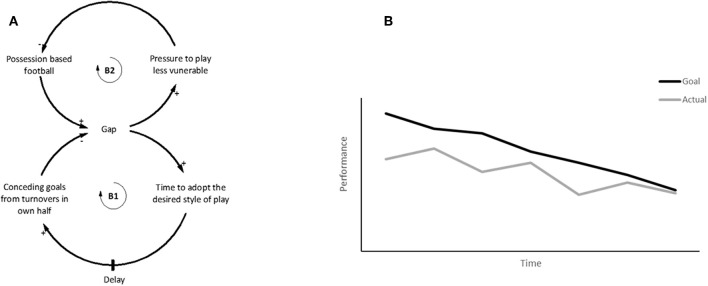
**(A)** The goal of the system is to play possession-based football, yet during actual performance teams are conceding goals from losing possession. Instead of allowing time and providing appropriate training the goal is lowered to play a less vulnerable style that moves away from the intended goal of the curriculum. **(B)** Conceptual behavior over time graph shows that continually lowering the goal will better align to current performance, however over time performance continues to degrade. Adapted from Braun (2002).

The Drifting goals archetype can be seen in youth football coaching (Figure 9A). For example, the goal of the national football curriculum in Australia is for youth teams to successfully build up attacks from the defence using a possession-based style of play (Berger, 2013). However, if players lack the technical and tactical ability to implement this tactic, possession can be easily lost in defensive locations of the pitch, leading to an increased number of turnovers and higher percentage shots on goal by opposing teams during the build-up phase of play. Although there is no empirical data on this phenomenon in youth football, the football SME contributing to the models described widespread occurrences of this issue. The SMEs described how poor results tended to encourage coaches to ignore the possession-based playing style advocated in the national curriculum, reverting instead to a less vulnerable playing style that requires less technical and tactical ability. As a result, the behavior over time of lowering the goal can lead to inadequate player development and poor performances, encouraging the goal to be continually lowered subsequently reducing overall performance (Figure 9B). By continually lowering the goal in this manner, it is possible that players are not developing the necessary skills to effectively build attacks from defence as intended by the curriculum.

#### Breaking the Drifting Goals Cycle

Critical to breaking the Drifting goals cycle is to determine what is initially driving the setting of the goals, and to assess the appropriateness of those goals (Braun, 2002). For example, it would be pertinent to ask whether the playing style characterized by possession based build up from defence, at all times, which was adopted from a top European footballing nation, suits the attributes and culture of Australian footballers and coaches? Although this example is focused on the Australian football environment which is familiar to the SMEs, the described SA may be generalizable globally. Patience to accomplish the desired style of play is required, integrated with appropriate design of training to facilitate the capability of the players to achieve the goal. This can be difficult as research suggests that coach education tends to demonstrate and provide examples of how to coach, but fails to provide an understanding of coaching principles required to develop expertise (Partington and Cushion, 2013). Similarly, a “one size fits all” approach is unlikely to suit the capabilities of all members of a given team, thereby neglecting the need to adapt coaching approaches to the needs of the individual (Renshaw et al., 2015). In addition, coaches could use objective measures to determine the level of performance in relation to the intended goal to assess performance over time, which would potentially identify any drifting of goals. Popular views in youth football are that the focus should be on development rather than results, which will potentially require a change in the mental models of coaches, players, administrators, and parents to understand that lowering the goal to achieve a fast fix, will have a negative impact on overall performance. Approaching this sensitive issue from a systems thinking perspective reduces the blame typically placed on players and coaches, and instead identifies the factors in the system that are contributing to the misalignment of the goals and actual performance.

#### Success to the Successful

**Figure 10:**
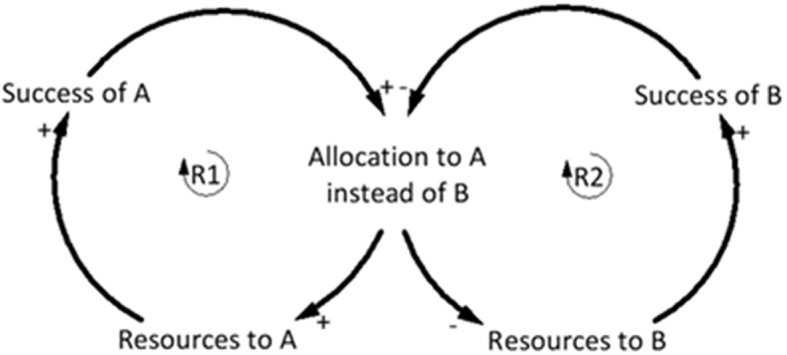
Generic “Success to the successful” archetype. Adapted from Braun (2002).

The Success to the successful archetype has been used across multiple domains to demonstrate that initial conditions of a system often dictate future performance of individuals within it (Kim, 1994). This archetype suggests that success is as dependent on the system structure as much as it is on talent (Kim, 1993; Braun, 2002). Based on the assumption of equal talent between two people, the reinforcing loop (R1) demonstrates that if person A is provided with more resources, the chance of success is increased compared to person B (Figure 10). The initial success of A justifies the provision of extra resources compared to B, but as B gets fewer resources, the success diminishes, further justifying additional resources to A (Senge, 1990; Kim, 1993; Braun, 2002) (Figure 10). Similar to the theory building aim of the Drifting goals archetype, the goal of Success to the successful archetype is to encourage a change in organizational theory to move the structure in a different direction (Kim, 1994).

The Success to the successful archetype is evident in talent identification and development in youth football, where early success is rewarded with increased resources including better coaching, support, and facilities. This archetype is typically driven by pressure on clubs and coaches to obtain immediate success, driven by results. The relative age effect (RAE) in sport is one example of many different factors (see review by Sarmento et al., 2018) that contributes to the Success to the successful archetype. The RAE suggests that players with advanced cognitive and physical maturation due to being born early in the competition year (compared to those born later), are more likely to be selected based upon this maturity (Deprez et al., 2013). These players may be provided with additional opportunities and resources relative to their later-born counterparts. Research from top footballing nations such as Belgium, England, Spain, Germany, France, and Italy has found that the RAE influences early identification and selection (Helsen et al., 2005). Whilst early success should be granted to those whom perform well initially, it is the structure of the identification and development system that could be restructured so that promising youth players, who given the appropriate resources could become elite, are not lost to football.

#### Breaking the Success to the Successful Cycle

**Figure 11:**
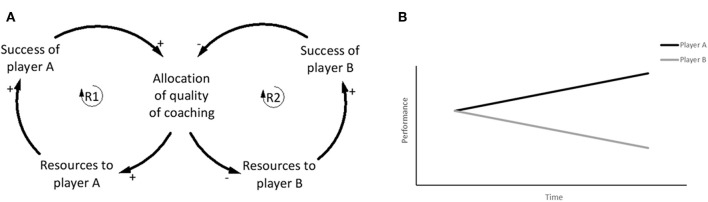
**(A)** The initial success of player A results in increased resources and better coaching, whereas player B who did not have initial success is not granted the same resources. The improvement of player B is less, which justifies the provision of even more resources to player A. **(B)** Behavior over time graph shows how the performance of the two players drifts apart from a similar starting point as resources are diverted to player A at the expense of player B. The continued success of Player A justifies the allocation of increased resources compared to player B. Adapted from Braun (2002).

The structure of the youth talent identification and development system is set up to force players to compete for limited resources in pursuit of the goal of developing elite players. There is a need to change the structure of the system so that players are not competing for limited resources in a system that creates few winners, and where talented players are not under-resourced due to the systemic structure. In the Success to the successful structure, one loop (R1) is set up to encourage early success (RAE as one example) (Figure 11A), whereas the appropriate leverage point to enact change to the structure of the system potentially lies in (R2), which reinforces a lack of resources for players continuing in this loop (Figure 11A). If the goal of the broader system is to identify and develop talented footballers, then (R2) is where the largest change to the system can be enacted. The consequences of such an intervention would potentially increase the depth of talent for clubs, and subsequently the depth of national teams, especially in developing and smaller football nations where the talent pool is limited. The behavior over time graph indicates the separation in performance as a result of player A receiving the initial resources (Figure 11B).

## Conclusion

The aim of this article was to demonstrate how SAs, commonly applied to explain and optimize system behavior in other domains, can be used to describe and respond to common recurring systemic problems in coaching in football. Although this article focused on coaching in football, the SAs could be applied at any level (e.g., club, organization, federation) and in any sport, and to numerous problems that may be impacting performance (e.g., the system structures and dynamics underpinning sports injury prevention). As such, the application of SAs in sport opens various new lines of inquiry for understanding and enhancing sports system performance in different contexts. The intention of this article is not to describe the numerous and intricate details of potential coaching problems, but to take a step back and appreciate the broader view of what the structure of the system can tell us about recurring issues associated with coaching in football. In doing so, practical implications can be extracted. A common theme which emerged across the SAs was that systemic problems often arise in football when the myriad of factors underpinning poor performance are not fully understood, and quick fixes are attempted as a result. Instead of the typical application of symptomatic solutions, fundamental solutions to the systemic problems should be sought based on a full understanding of the problem. Furthermore, there is a need for a shared understanding that there will be some delay post implementation of the appropriate corrective action, before problems are reduced or eradicated. This requires a paradigm shift in the mental models of coaches, coach educators, club senior executives, and key club stakeholders.

The SAs presented provide practical templates of common coaching issues that can be used to prompt discussions around how to avoid ineffective interventions and instead make sustainable improvements across multiple aspects of football performance and coaching. The SAs could be used in coaching and in sports management education curriculums to demonstrate how a systems thinking approach can benefit users by understanding how system structures influence both the behavior of the system and the actors within that system.

## Author Contributions

SM and PS: concept. SM, GR, AH, KD, AG, CS, and PS: performed the analysis and writing and revision of article.

### Conflict of Interest

The authors declare that the research was conducted in the absence of any commercial or financial relationships that could be construed as a potential conflict of interest.
